# Novel In Vitro Investigational Methods for Modeling Skin Permeation: Skin PAMPA, Raman Mapping

**DOI:** 10.3390/pharmaceutics12090803

**Published:** 2020-08-25

**Authors:** Stella Zsikó, Erzsébet Csányi, Anita Kovács, Mária Budai-Szűcs, Attila Gácsi, Szilvia Berkó

**Affiliations:** Institute of Pharmaceutical Technology and Regulatory Affairs, Faculty of Pharmacy, University of Szeged, Eötvös u. 6, H-6720 Szeged, Hungary; zsiko.stella@pharm.u-szeged.hu (S.Z.); csanyi@pharm.u-szeged.hu (E.C.); anita.kovacs@pharm.u-szeged.hu (A.K.); maria.szucs@pharm.u-szeged.hu (M.B.-S.); gacsi.attila@pharm.u-szeged.hu (A.G.)

**Keywords:** skin PAMPA, Raman mapping, Franz cell, drug release, skin permeation, in vitro release test (IVRT), in vitro permeation test (IVPT)

## Abstract

The human skin is marked as a standard by the regulatory agencies in the permeation study of dermal formulations. Artificial membranes can substitute human skin to some extent. Academicians and pharmaceutical corporations are focusing their efforts on developing standardized protocols and safe, reliable options to substitute human skin for carrying out permeability studies. Our research aim was to study the applicability of new techniques in the case of different types of dermal formulations. The skin parallel artificial membrane permeability assay (PAMPA) method and Raman mapping were compared to the gold-standard Franz cell method. A hydrogel and two types of creams were investigated as the most generally used dermal preparations. The values of the diffused drug were closer to each other in PAMPA and Franz cell measurement. The diffused amount of drug showed the same order for the different formulations. These results correlate well with the results of Raman mapping. Our conclusions suggest that all early screening examinations can be performed with model tools such as skin PAMPA supplemented with methods like Raman mapping as a semi-quantitative method.

## 1. Introduction

The growing number of dermal formulations requires the development of test methods, in particular appropriate in vitro drug permeation tests. There are some in vitro models which are widely used in skin permeation studies, but there is a demand for more rapid and cost-effective studies that better model the real conditions. Permeation tests are usually made to detect the amount of drug permeated through the skin (or skin mimic membrane) over time in connection with the diffusion area and may provide information on drug release, interactions and mechanisms of drug permeation. In the latest European Medicines Agency (EMA) document (Draft Guideline on Quality and Equivalence of Topical Products) Franz diffusion cell tests are classified as follows: in vitro release tests (IVRT) and in vitro permeation tests (IVPT). One of the greatest differences between IVRT and IVPT tests is the membrane used. In the case of IVRT the membrane is synthetic (lipid-based or non-lipid based model membranes), while in the case of IVPT the membrane is biological (e.g., human epidermis). An IVRT using diffusion cells evaluates the rate and extent of release of an active substance from the proposed formulation. An IVPT is used to characterize the permeation profile of the drug, which is an acceptable permeation kinetic test. In the early stages of development, IVRT should be used, thereafter, IVPT can be used for promising formulations [1]. 

The vertical Franz diffusion cell is generally used for modelling the release and permeation of drugs in the case of dermal drug delivery systems. This device consists of a cell that holds a chamber for drug application, a membrane (it can be synthetic and biological, too) within which the drug may diffuse, and an acceptor media chamber from which samples may be examined [2,3,4,5,6,7]. The most relevant membrane for assessing the in vitro permeation of drugs is the human skin. Excised human skin layer (epidermis) is the gold standard. The availability of human skin is limited, so artificial membranes are often used [4,8,9,10]. However, it has been found that simple artificial membranes do not match the characteristics of the skin and, consequently, their use is suggested only in the determination of drug release. Therefore, there is a growing demand for new artificial membranes that model the human skin well to provide relevant and useful results in predicting the permeation of the drug from the dermal drug delivery preparations [1,11]. 

Artificial skin surrogates have a long history. Nowadays, more and more complex membrane systems have become available. When porous filters are loaded with lipids, these substitutes are more similar to skin attributes [12]. Based on the parallel artificial membrane permeability assay technique (PAMPA), which has been used successfully to predict gastrointestinal and blood-brain barrier absorption, Ottaviani et al. described the use of a skin PAMPA first. This skin PAMPA membrane is filled with an optimized mixture of silicone (70%) and isopropyl myristate (30%) to reduce skin permeability [13]. Recently a novel skin PAMPA model has become available where the membrane contains the special components of the skin barrier such as cholesterol, free fatty acid, and a ceramide-analogue compound which imitates the features of skin lipid matrix [14,15]. This PAMPA is specifically designed for modelling permeation through the skin. It is a 96-well plate-based method so it is a fast, low-cost and high-throughput method. So far, there have been few measurement results, but they are encouraging [16,17,18,19,20,21,22].

In addition to permeation studies through different membranes, the Raman microscopy method has been developed as an important technique for a better understanding of skin structure and drug delivery via human skin [23]. Raman spectroscopy can give information about the spatial distribution of the drug in the skin layers based on the characteristic vibrational energy levels of the structure of a molecule. This semi-quantitative technique is suitable for monitoring the permeation of exogenous materials into the skin layers [24,25,26,27,28].

Currently, skin PAMPA is not listed in the official recommendations of skin permeation methods but Raman spectroscopy can be used [1]. Skin PAMPA is a relatively new method still under development. In the future, if there is enough favorable investigation, it might be recommended by authorities. Confocal Raman spectroscopy is not sufficiently established to provide pivotal equivalence data but it may be supportive; thus, research of this area is very important in order to broaden the available methods for more predictable investigation of skin permeation. 

The aim of this research was to investigate the applicability of skin PAMPA and Raman mapping methods compared with the widely used gold-standard Franz cell method. The most commonly used dermal preparations such as a hydrogel and two types of creams (containing diclofenac sodium, DFNa) were investigated.

## 2. Materials and Methods 

### 2.1. Materials

Diclofenac sodium, ethanol, cetyl-stearyl alcohol, liquid paraffin, white petrolatum, microbiological preservative, beeswax, wool fat, oleyl oleate and castor oil were obtained from Hungaropharma Ltd. (Budapest, Hungary). Polysorbate 60 was obtained from Sigma-Aldrich (Budapest, Hungary). Methocel^TM^ E4M (hydroxypropyl methylcellulose) was purchased from Colorcon (Budapest, Hungary). The water used has been filtered and deionized using the Millipore (Milford, MA, USA) Milli-Q method. The skin PAMPA sandwiches (P/N: 120657), hydration solution (P/N: 120706) and stirring bars (P/N: 110066) were purchased from Pion, Inc. (Woburn, MA, USA). The UV plates were from Greiner Bio-one (Kremsmünster, Austria). UV-star microplate has been used (transparent, flat bottom, half area). The cellulose acetate filter (Porafil membrane filter, cellulose acetate, pore diameter: 0.45 µm) was purchased from Macherey-Nagel GmbH & Co. KG (Düren, Germany). Excised human skin was collected by routine plastic surgery treatment from a Caucasian female patient at the Department of Dermatology and Allergology, Szeged University. The in vitro skin permeation procedure does not require ethical approval or consent of the patient in compliance with Health Act CLIV of 1997, Section 210/A in Hungary. The Ethical Committee of the University of Szeged, Albert Szent-Györgyi Clinical Center was informed about the investigations (Human Investigation Review Board license number: 83/2008).

### 2.2. Methods

#### 2.2.1. Sample Preparations

The conventional hydrogel was prepared with 1% (*w/w*) DFNa dissolved in the mixture of purified water (65.5%) and ethanol (30%). Methocel E4M (3%) and microbiological preservative (0.5%) were added. In the case of the *o*/*w* cream, the oily phase consisting of cetyl-stearyl alcohol (4%), liquid paraffin (12%), white petrolatum (20%) and polysorbate 60 (4%) was heated up to 60 °C. Then, hot water (57%) was added to the oily phase under agitation. DFNa (1%) was dispersed in the preparation and it was homogenized until the mixture was cooled. Finally, the microbiological preservative (2%) was added. In the case of the *w*/*o* cream, the oily phase consisting of beeswax (10%), wool fat (10%), oleyl oleate (5%) and castor oil (40%) was heated up to 60 °C. Then, hot water (29%) was added to the oily phase under agitation. Finally, DFNa (1%) was dispersed in the preparation and it was homogenized. 

#### 2.2.2. Drug Release and Permeation Studies

A vertical Franz type diffusion cell (Logan Automated Dry heat sampling system, Logan Instruments Corporation, NJ, USA) was used to model the drug release from the formulations through a synthetic membrane (IVRT) and permeation through human heat-separated epidermis (IVPT). As the synthetic membrane, 0.45 µm cellulose membrane (Porafil membrane filter, cellulose acetate) was used. The heat-separated human epidermis (HSE) was prepared with the following method: the excised human subcutaneous fat-free skin was placed in a water bath (60 ± 0.5 °C, 1 min), and the epidermis was separated from the dermis. A quantity of 0.300 g of the sample was placed on the membrane in the donor chamber. Thermostated phosphate buffer solution (PBS pH 7.4 ± 0.15), made in-house, kept at 32 ± 0.5 °C was used as the receptor phase which was 9 mL. The investigation lasted for 6 h (sampling times: 0.5; 1; 2; 3; 4; 5; 6 h. A Thermo Scientific Evolution 201 spectrometer with a Thermo Insight v1.4.40 software kit (Thermo Fisher Scientific, Waltham, MA, USA) at a wavelength of 275 nm was used to assess the DFNa concentration in the receptor solution.

The skin PAMPA sandwiches were used after 24 h hydration time. A special hydration solution (Pion, Inc., Billerica, MA, USA) was used. As donor phase, 70 µL of the preparations were used in all well of the formulation plate. As receptor phase, phosphate buffer solution (PBS pH 7.4 ± 0.15) was used. The top plate was loaded with a fresh receptor solution of 250 μL, and a stirring bar was also used in each well. The Gut-Box™ from Pion, Inc. was used for stirring; the sandwich was incubated at 32 °C. The receptor solution was analyzed after 0.5, 1, 2, 3, 4, 5 and 6 h of incubation [21]. The quantity of DFNa was measured with UV spectroscopy at 275 nm using a SPECTROstarNano UV plate reader by BMG LABTECH GmbH (Ortenberg, Germany).

Permeation profiles of dermal formulations were obtained. The cumulative amount (Q) of DFNA penetrated per cm^2^ at 6 h was calculated. The flux (J) was the slope of the cumulative amounts of DFNa (µg/cm^2^) permeated versus time (h) profiles. Time point correlations between the amounts of drug penetrated through heat-separated human epidermis and skin PAMPA membrane were shown and correlation coefficients (R^2^) were calculated. 

#### 2.2.3. Investigation of Skin Permeation with Raman Microscopy

Excised human subcutaneous fat-free skin (epidermis and dermis) was used. It was obtained from a Caucasian female patient who underwent abdominal plastic surgery. 1 cm^2^ of the skin surface was treated with the formulations for 3 h at 32 °C. The treated skins were frozen and sectioned (10-μm-thick cross-sections) with a Leica CM1950 cryostat (Leica Biosystems GmbH, Wetzlar, Germany). The microtomed skin samples were placed on an aluminum surface with the SC towards the top of the plate. Raman spectroscopic measurements were made with a Thermo Fisher DXR Dispersive Raman Spectrometer (ThermoFisher Scientific Inc., Waltham, MA, USA) equipped with a CCD camera and a diode laser. A laser light source of 780 nm wavelength was used, with a maximum power of 24 mW, which is the best source for studying biological samples, presenting sufficient energy for the vibrations of protein alternatives in the skin. With the use of this type of laser source, fluorescence had less effect. The microscopic lens used for the measurements was magnified by 50 × and the pinhole aperture was 25 μm. A 200 to 1.800 μm area was explored in the case of chemical mapping; the step size was 50 μm vertically and horizontally. 205 spectra were observed, 16 scans were reported to accumulate each spectrum, and the exposure period was 2 s. When analyzing the treated vs. untreated skin samples, the different spectra of each component of the formulations and diclofenac sodium were used as reference points. A laser light of 532 nm was used to record the different spectra of the components and formulations. 32 scans were registered for each spectrum, with an exposure time of 6 s. The optics magnitude in the Raman microscope was 10 with a 25 μm slit aperture. Data acquisition and analysis were accomplished using OMNICTM8.2 for Dispersive Raman software package (ThermoFisher Scientific Inc., Waltham, MA, USA) [24,27]. 

#### 2.2.4. Statistical Analysis

Statistical data analysis was performed using Prism for Windows software (GraphPad Software Inc., La Jolla, CA, USA). The values were compared using two-way ANOVA followed by the Bonferroni test. A level of *p* ≤ 0.05 was taken as significant, *p* ≤ 0.01 as very significant, and *p* ≤ 0.001 as highly significant.

## 3. Results and Discussion

In the case of dermal permeations, there are three main parameters which influence the effect: the characteristics of the active ingredient, the properties of the formulation and the condition of the skin [29,30]. From among the three main parameters, the properties of the preparation were changed in this study, and the measurements were performed with Franz cell, skin PAMPA and Raman spectroscopy. Three most commonly used dermal formulations were compared: a hydrogel and *o*/*w* and *w*/*o* creams. 

### 3.1. Comparision Study of Franz Cell and Skin PAMPA

**Figure 1:**
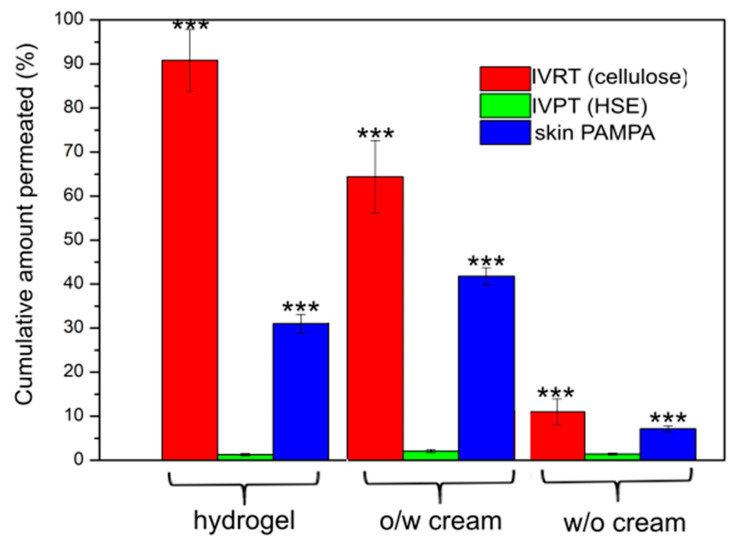
Cumulative amount permeated at the end of 6 h (%). *** *p* < 0.001 vs. HSE.

The Franz cell method and the skin PAMPA methods are based on the quantitative measurement of drug permeation through a skin-mimicking membrane. Figure 1. shows the amount of drug passing through the different membranes in 6 h in percentage from the different types of formulations. In the IVRT measurement, most of the DFNa was released from the hydrogel within 6 h, followed by the *o*/*w* cream, and the least drug was detectable from the *w*/*o* cream. In the IVPT test (gold-standard method), significantly less drug was delivered to the receptor chamber compared to IVRT, and if the formulations were aligned, different result was obtained. The most diffused active ingredients were detected with the *o*/*w* cream, followed by the hydrogel and the least with the *w*/*o* cream. This is due to the barrier function of the skin, which is primarily responsible for the stratum corneum cell layer. It is important to know the extent of release (IVRT), but this does not provide relevant information for penetration. It is clear that it is very important to examine the permeation process through the skin to know not only the released amount of drug, but also to see the interactions of the drug or the drug delivery system with the skin.

The results of the skin PAMPA method were compared to the results of the IVPT and IVRT methods. The detected DFNa values were closer to the values measured on HSE than the data from the IVRT tests. The diffused amount of drug from the different formulations showed the same order for both the IVPT and the skin PAMPA methods: *o*/*w* cream > hydrogel > *w*/*o* cream. In the case of skin PAMPA, the SD values were lower compared with IVPT, where the variability and differences of the human skin membrane are higher. 

In addition to the order, it can be concluded that significantly more DFNa penetrated through the cellulose membrane and skin PAMPA vs. HSE, however, the results of skin PAMPA are closer to the results of HSE. 

The mathematical evaluation of the results is shown in Table 1. Permeation parameters (Q, and J) show the differences between the methods and formulations. There were significant differences between the formulations, so it is clear that all methods have good sensitivity to show significant differentiation between the different formulations, so it is a very good tool in the preformulation phase to find the best suited one for the purpose of use. A comparison of the results indicates that the skin PAMPA method was closer to the gold standard (IVPT) method. Its use before IVPT tests may be beneficial as it shows differences between formulations in the same way as HSE. Thus, in pharmaceutical developments, less effective preparations can be excluded quickly and cheaply from the many formulations.

**Figure 2:**
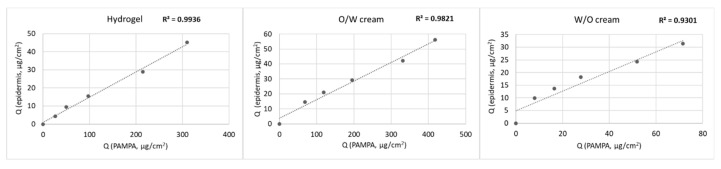
Diclofenac sodium permeation, time point correlations between the amounts of drug penetrated through heat-separated human epidermis and skin PAMPA membrane.

Figure 2 shows the correlation of the skin PAMPA membrane to HSE. The skin PAMPA membrane demonstrates high correlation to HSE in this study. The time point correlation between skin PAMPA and HSE was in the range of 0.93–0.99. 

### 3.2. RAMAN Mapping

The Raman correlation map proves the presence of the penetrated drug formulations in the different regions of the human skin, from the skin surface to the lower layers of the dermis after the treatment with the different compositions. The Raman spectra of the skin are really diverse and consist of numerous bands originating from different skin segments (e.g., nucleic acids, lipids, proteins) [27,31,32]. Several bands are overlapping with the spectra of the examined preparations. During the Raman experiments, the differences in the localization in the skin regions of the formulations were determined and compared with the Franz cell and skin PAMPA results. 

The correlation maps, which showed the distribution of DFNa, were produced by fitting the appropriate spectra to the spectra of the treated skin. DFNa is easily determined from the formulations but the intensities of the characteristic DFNa peaks are very low. Therefore, the spectra of the pure API could not be used to make an acceptable correlation map. In this case, we had to use the spectrum of the whole preparation to make the skin distribution correlation maps, which indicates the presence of DFNa as well. The spectral maps were resolved in order to verify the presence of the formulation in the different regions of the human skin. The fingerprint region of the preparation spectra was related to the spectra of human skin spectra being tested and untreated. The similarity was shown as intensity. The distribution profiles describing the relationship between the map spectra (treated skin specimen) and the defined reference spectrum (fingerprint region) were created. The resulting correlation intensity values of the map spectra are similar to the match values of the reference spectra. A more powerful intensity rate means a higher correlation with the reference spectrum.

**Figure 3:**
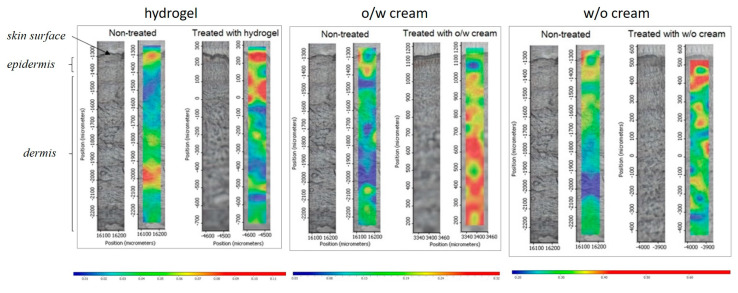
Raman correlation maps for the distribution of diclofenac sodium in human skin specimens after treatment with hydrogel, o/w cream and w/o cream. Untreated skin is also displayed as a control in all cases. Color coding of drug formulation content: red > yellow > green > blue.

The Raman chemical maps of the preparations are shown in Figure 3. In the case of the hydrogel, the most penetrated drugs are found in the upper layers of the skin, the epidermis and the upper dermis. The oil-in-water cream was most penetrated into the deeper layers of the skin. This is due to the emulsifier, which increases permeation. In the case of the water-in-oil cream, most of the composition could be found only in the stratum corneum region, and deeper permeation was blocked. This is due to its really high oil content in the external phase, which cannot pass through the hydrophilic layer of the epidermis.

These results correlate well with the results of IVPT and skin PAMPA. In correlation with these results, the *o*/*w* cream shows the most effective permeation results where the formulation could be found in the dermis, followed by hydrogel, where the formulation passed through the regions of the epidermis and dermis. The permeation of the *w*/*o* cream was the lowest with all the methods during the time of the experiment.

In the case of IVRT measurements, the hydrogel showed the highest drug release values. The hydrogel is an aqueous-based system where the drug is in a dissolved form, and diffusion through the synthetic membrane is high. The highest permeation from the o/w cream was observed in the IVPT and Skin PAMPA measurements because DFNa is in the outer aqueous phase of the cream, and the emulsifier content of the cream promotes penetration through human skin and special skin-mimicking membrane. The release and permeation of DFNa from the w/o cream were extremely low in all measurements. The drug is presumably in the inner phase of the cream, and the low diffusion and penetration can be explained by the fact that the diffusion of DFNa through the oil phase limits the release of the drug.

## 4. Conclusions

The skin PAMPA method was investigated to prove the applicability in skin permeation tests. This method presented high correlations with the IVPT method compared to different dermal formulations. The ranking of the penetrated drug from different formulations was the same, but the permeability rates were different. The skin PAMPA system seems to be an ideal test before IVPT using human skin with a defined thickness. The advantages of PAMPA method are as follows: it is easy to use and store, comparatively low-cost, inert, and gives reproducible results. The main disadvantage of this method is that the measurement time is usually limited (6 h).

This work also highlights the capability of Raman spectroscopy as a nondestructive technique for studying skin distribution and following the active ingredient in the skin layers. It could estimate the relative amounts of preparations penetrated into the different skin layers semi-quantitatively. It is important to understand how different formulations influence the permeation of active agents into/through the skin as this presents relevant information for formulation developers. Like all measurement methods, Raman mapping has its drawbacks. It is difficult to reproduce, expensive lasers are needed, only qualitative information can be extracted, the measurement time is usually long, and not all active substances can be tested with Raman. Its use is recommended for formulations that have previously performed well on IVRT and IVPT tests. Currently, only qualitative (or semi-quantitative) determinations are possible with Raman mapping, but thanks to continuous improvements, quantitative evaluations may also be feasible in the future [25,33,34].

In the current study, the results of Raman mapping have high correlation with the results of skin PAMPA and IVPT methods. Based on our results, the conclusions suggest that all early screening examinations can be performed with model tools such as IVRT and skin PAMPA using cheaper synthetic membranes, and these final formulations should be examined with IVPT to collect permeation data supplemented with qualitative methods like Raman mapping using human skin.

In conclusion, this study has proved that the new skin PAMPA method and the Raman mapping technique have the potential to be used as a screening tool to choose the best dermal formulation in the pharmaceutical, cosmetic, and personal care industries. Future studies in this field are still needed to examine and compare the different methods. Other types of formulations and active substances should also be tested. It would also be useful to study different drug concentrations for the same formulations to support the suitability of the method for monitoring dermal penetration. The development of methodologies to improve the throughput of formulation testing will help to promote a better understanding of preparation variables and mechanisms of skin permeation. Therefore, it is essential to highlight that the investigated in vitro models provide important tools for screening a series of drug formulations, evaluation of skin permeation and mechanism of action of the carrier systems, and evaluation of rank of skin transport.

## Figures and Tables

**Table 1:** Permeation parameters of diclofenac sodium through cellulose membrane, heat-separated human epidermis and skin parallel artificial membrane permeability assay (PAMPA) membrane after 6 h.

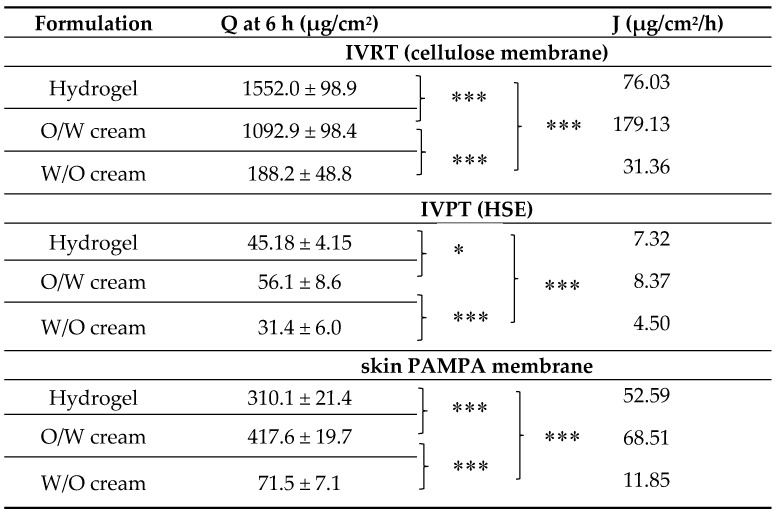

**Q**, cumulative amount of diclofenac sodium penetrated per cm^2^ at 6 h (mean ± SD, *n* = 6); **J**, flux determined from the slope of the cumulative amounts of diclofenac sodium permeated (µg/cm^2^) versus time (h) profiles; * *p* < 0.05, ***, *p* < 0.001.

## References

[1] (2018). Draft Guideline on Quality and Equivalence of Topical Products.

[2] Ruela A.L.M., Perissinato A.G., Lino M.E., Mudrik P.S., Pereira G.R. (2016). Evaluation of skin absorption of drugs from topical and transdermal formulations. Braz. J. Pharm. Sci..

[3] Hauck W.W., Shah V.P., Shaw S.W., Ueda C.T. (2007). Reliability and Reproducibility of Vertical Diffusion Cells for Determining Release Rates from Semisolid Dosage Forms. Pharm. Res..

[4] OECD (2004). Test Guideline 428: Skin Absorption: In Vitro Method.

[5] Buist H., Craig P., Dewhurst I., Hougaard Bennekou S., Kneuer C., Machera K., Pieper C., Court Marques D., Guillot G., European Food Safety Authority (EFSA) (2017). Guidance on dermal absorption. EFSA J..

[6] Moser K., Kriwet K., Naik A., Kalia Y.N., Guy R.H. (2001). Passive skin penetration enhancement and its quantification in vitro. Eur. J. Pharm. Biopharm..

[7] Zsikó S., Csányi E., Kovács A., Budai-Szűcs M., Gácsi A., Berkó S. (2019). Methods to Evaluate Skin Penetration in Vitro. Sci. Pharm..

[8] Machado A.C.H.R., Lopes P.S., Raffier C.P., Haridass I.N., Roberts M., Grice J., Leite-Silva V.R. (2017). Skin Penetration. Cosmetic Science and Technology.

[9] OECD (2011). Guidance Notes on Dermal Absorption, Series on Testing and Assessment No. 156.

[10] Abd E., Yousuf S., Pastore M., Telaprolu K., Mohammed Y., Namjoshi S., Grice J., Roberts M. (2016). Skin models for the testing of transdermal drugs. Clin. Pharmacol. Adv. Appl..

[11] Neupane R., Boddu S.H.S., Renukuntla J., Babu R.J., Tiwari A.K. (2020). Alternatives to Biological Skin in Permeation Studies: Current Trends and Possibilities. Pharmaceutics.

[12] Flaten G.E., Palac Z., Engesland A., Filipović-Grčić J., Vanić Ž., Škalko-Basnet N. (2015). In vitro skin models as a tool in optimization of drug formulation. Eur. J. Pharm. Sci..

[13] Ottaviani G., Martel S., Carrupt P.-A. (2006). Parallel Artificial Membrane Permeability Assay: A New Membrane for the Fast Prediction of Passive Human Skin Permeability. J. Med. Chem..

[14] Sinkó B., Kökösi J., Avdeef A., Takács-Novák K. (2009). A PAMPA Study of the Permeability-Enhancing Effect of New Ceramide Analogues. Chem. Biodivers..

[15] Sinkó B., Garrigues T.M., Balogh G.T., Nagy Z.K., Tsinman O., Avdeef A., Takács-Novák K. (2012). Skin–PAMPA: A new method for fast prediction of skin penetration. Eur. J. Pharm. Sci..

[16] Sinkó B., Vizserálek G., Takács-Novák K. (2015). Skin PAMPA: Application in practice. ADMET DMPK.

[17] Vizserálek G., Berkó S., Tóth G., Balogh R., Budai-Szűcs M., Csányi E., Sinkó B., Takács-Novák K. (2015). Permeability test for transdermal and local therapeutic patches using Skin PAMPA method. Eur. J. Pharm. Sci..

[18] Balázs B., Vizserálek G., Berkó S., Budai-Szűcs M., Kelemen A., Sinkó B., Takács-Novák K., Szabó-Révész P., Csányi E. (2016). Investigation of the Efficacy of Transdermal Penetration Enhancers through the Use of Human Skin and a Skin Mimic Artificial Membrane. J. Pharm. Sci..

[19] Luo L., Patel A., Sinko B., Bell M., Wibawa J., Hadgraft J., Lane M.E. (2016). A comparative study of the in vitro permeation of ibuprofen in mammalian skin, the PAMPA model and silicone membrane. Int. J. Pharm..

[20] Zhang Y., Lane M.E., Hadgraft J., Heinrich M., Chen T., Lian G., Sinko B. (2019). A comparison of the in vitro permeation of niacinamide in mammalian skin and in the Parallel Artificial Membrane Permeation Assay (PAMPA) model. Int. J. Pharm..

[21] Zsikó S., Cutcher K., Kovács A., Budai-Szűcs M., Gácsi A., Baki G., Csányi E., Berkó S. (2019). Nanostructured Lipid Carrier Gel for the Dermal Application of Lidocaine: Comparison of Skin Penetration Testing Methods. Pharmaceutics.

[22] Martins P.P., Estrada A.D., Smyth H.D.C. (2019). A human skin high-throughput formulation screening method using a model hydrophilic drug. Int. J. Pharm..

[23] Dragicevic N., Maibach H.I. (2017). Percutaneous Penetration Enhancers Drug Penetration into/through the Skin.

[24] Berkó S., Zsikó S., Deák G., Gácsi A., Kovács A., Budai-Szűcs M., Pajor L., Bajory Z., Csányi E. (2018). Papaverine hydrochloride containing nanostructured lyotropic liquid crystal formulation as a potential drug delivery system for the treatment of erectile dysfunction. Drug Des. Devel. Ther..

[25] Franzen L., Selzer D., Fluhr J.W., Schaefer U.F., Windbergs M. (2013). Towards drug quantification in human skin with confocal Raman microscopy. Eur. J. Pharm. Biopharm..

[26] Mao G., Flach C.R., Mendelsohn R., Walters R.M. (2012). Imaging the Distribution of Sodium Dodecyl Sulfate in Skin by Confocal Raman and Infrared Microspectroscopy. Pharm. Res..

[27] Bakonyi M., Gácsi A., Kovács A., Szűcs M.-B., Berkó S., Csányi E. (2018). Following-up skin penetration of lidocaine from different vehicles by Raman spectroscopic mapping. J. Pharm. Biomed. Anal..

[28] Pyatski Y., Zhang Q., Mendelsohn R., Flach C.R. (2016). Effects of permeation enhancers on flufenamic acid delivery in Ex vivo human skin by confocal Raman microscopy. Int. J. Pharm..

[29] Machado M., Hadgraft J., Lane M.E. (2010). Assessment of the variation of skin barrier function with anatomic site, age, gender and ethnicity: Assessment of the variation of skin barrier function. Int. J. Cosmet. Sci..

[30] Jepps O.G., Dancik Y., Anissimov Y.G., Roberts M.S. (2013). Modeling the human skin barrier—Towards a better understanding of dermal absorption. Adv. Drug Deliv. Rev..

[31] Synytsya A., Alexa P., Besserer J., De Boer J., Froschauer S., Gerlach R., Loewe M., Moosburger M., Obstová I., Quicken P. (2004). Raman spectroscopy of tissue samples irradiated by protons. Int. J. Radiat. Biol..

[32] Ilchenko O., Pilgun Y., Makhnii T., Slipets R., Reynt A., Kutsyk A., Slobodianiuk D., Koliada A., Krasnenkov D., Kukharskyy V. (2016). High-speed line-focus Raman microscopy with spectral decomposition of mouse skin. Vib. Spectrosc..

[33] Franzen L., Windbergs M. (2014). Accessing Raman spectral variability in human stratum corneum for quantitative in vitro depth profiling: Raman spectral variability in human stratum corneum. J. Raman Spectrosc..

[34] Pezzotti G., Boffelli M., Miyamori D., Uemura T., Marunaka Y., Zhu W., Ikegaya H. (2015). Raman spectroscopy of human skin: Looking for a quantitative algorithm to reliably estimate human age. J. Biomed. Opt..

